# Molecular Ghrelin System in the Pancreatic Acinar Cells: The Role of the Polypeptide, Caerulein and Sensory Nerves

**DOI:** 10.3390/ijms18050929

**Published:** 2017-05-02

**Authors:** Joanna Bonior, Piotr Ceranowicz, Ryszard Gajdosz, Beata Kuśnierz-Cabala, Piotr Pierzchalski, Zygmunt Warzecha, Artur Dembiński, Michał Pędziwiatr, Michalina Kot, Anna Leja-Szpak, Katarzyna Nawrot-Porąbka, Paweł Link-Lenczowski, Rafał Olszanecki, Krzysztof Bartuś, Jolanta Jaworek

**Affiliations:** 1 Department of Medical Physiology, Faculty of Health Sciences, Jagiellonian University Medical College, 12 Michałowskiego St., 31-126 Krakow, Poland; joanna.bonior@uj.edu.pl (J.B.); piotr.pierzchalski@uj.edu.pl (P.P.); m.kot@uj.edu.pl (M.K.); a.leja-szpak@uj.edu.pl (A.L.-S.); k.nawrot-porabka@uj.edu.pl (K.N.-P.); p.link-lenczowski@uj.edu.pl (P.L.-L.); jolanta.jaworek@uj.edu.pl (J.J.); 2 Department of Physiology, Faculty of Medicine, Jagiellonian University Medical College, 16 Grzegórzecka St., 31-531 Krakow, Poland; mpwarzec@cyf-kr.edu.pl (Z.W.); mpdembin@cyf-kr.edu.pl (A.D.); 3 Department of Emergency Medical Care, Faculty of Health Sciences, Jagiellonian University Medical College, 12 Michałowskiego St., 31-126 Krakow, Poland; ryszard.gajdosz@uj.edu.pl; 4 Department of Diagnostics, Chair of Clinical Biochemistry, Faculty of Medicine Jagiellonian University Medical College, 15 A Kopernika St., 31-501 Krakow, Poland; mbkusnie@cyf-kr.edu.pl; 5 2nd Department of Surgery, Faculty of Medicine, Jagiellonian University Medical College, 21 Kopernika St., 31-501 Krakow, Poland; michal.pedziwiatr@uj.edu.pl; 6 Department of Pharmacology, Faculty of Medicine, Jagiellonian University Medical College, 16 Grzegórzecka St., 31-531 Krakow, Poland; rafal.olszanecki@uj.edu.pl; 7 Department of Cardiovascular Surgery and Transplantology, Faculty of Medicine, Jagiellonian University, JP II Hospital, 80 Prądnicka St., 31-202 Krakow, Poland; krzysztof.bartus@uj.edu.pl

**Keywords:** ghrelin, growth hormone secretagogues receptor type 1a, caerulein, pancreatic acinar cells, acute pancreatitis, AR42J cells, sensory nerves

## Abstract

Ghrelin (GHRL) is an endogenous ligand for the growth hormone secretagogue receptor (GHS-R). Experimental studies showed that GHRL protects the stomach and pancreas against acute damage, but the effect of GHRL on pancreatic acinar cells was still undetermined. Aim: To investigate the effect of GHRL and caerulein on the functional ghrelin system in pancreatic acinar cells taking into account the role of sensory nerves (SN). Methods: Experiments were carried out on isolated pancreatic acinar cells and AR42J cells. Before acinar cells isolation, GHRL was administered intraperitoneally at a dose of 50 µg/kg to rats with intact SN or with capsaicin deactivation of SN (CDSN). After isolation, pancreatic acinar cells were incubated in caerulein-free or caerulein containing solution. AR42J cells were incubated under basal conditions and stimulated with caerulein, GHRL or a combination of the above. Results: Incubation of isolated acinar cells with caerulein inhibited GHS-R and GHRL expression at the level of mRNA and protein in those cells. Either in rats with intact SN or with CDSN, administration of GHRL before isolation of acinar cells increased expression of GHRL and GHS-R in those cells and reversed the caerulein-induced reduction in expression of those parameters. Similar upregulation of GHS-R and GHRL was observed after administration of GHRL in AR42J cells. Conclusions: GHRL stimulates its own expression and expression of its receptor in isolated pancreatic acinar cells and AR42J cells on the positive feedback pathway. This mechanism seems to participate in the pancreatoprotective effect of GHRL in the course of acute pancreatitis.

## 1. Introduction

Ghrelin (GHRL) molecule consists of 28 amino acids and a molecular mass of rat GHRL is 3314 Da. GHRL is formed from its 117-amino acid precursor: preproghrelin [1]. This precursor is encoded by the *ghrl* gene, present in all vertebrates, and in humans it is located on chromosome 3 in region 3p25–26. It has a length of 511 bp and consists of five exons and four introns [2]. The first exon contains only 20 base pairs, which encode a section that does not undergo translation. *Ghrl* gene has two transcription start sites, which leads to the expression of two different transcripts—A and B. The mature GHRL molecule is encoded by exons 1 and 2 [3,4]. The 5′ flanking region of the gene of human GHRL includes TATA box-like sequence (TATATAA; located at positions from −585 to −579) and is considered to be the binding site for a number of transcription factors [1].

Preproghrelin molecule consists of a 23-amino-acid signal sequence and the 94-amino-acid proghrelin. During the consecutive stage, the prohormone undergoes modification by GHRL *O*-acyltransferase (GOAT), specifically octanoylates Ser at position 3 of the polypeptide chain. Then, there is the process of limited proteolysis of the prohormone by PC1/3 protease at position Arg^28^/Ala^29^ to the 28-amino-acid N-terminal biologically active GHRL molecule [5,6,7,8,9]. From preproghrelin, apart from the GHRL, another hormone, obestatin is also created [5,6,7,8,9,10].

There are two main molecular forms of GHRL: acylated (AG) and unacylated (UAG) [1]. The AG form that occurs in humans is the 28-amino-acid peptide. The first natural hormone has been identified in the rat stomach, released from enteroendocrine cells, the so-called X/A-like cells, in which the hydroxyl group Ser-3 is acylated by an n-octane acid. This particular characteristic of the GHRL molecule constitutes the key element of its biological activity, i.e., its ability to pass the blood–brain barrier (BBB), and growth hormone (GH) secretagogue receptor (GHS-R) activation [1,11,12,13,14]. The UAG form is devoid of *N*-acyl radical and does not show receptor activity towards GHS-R1a, as well as towards releasing GH or other endocrine activity in rats [15,16,17,18]. The research to date demonstrated, however, that this non-esterificated GHRL form is also a biologically active molecule. This effect is produced by receptors other than GHS-R1a, and the activity is probably mediated by a not yet identified GHS-R [19]. Because its genome database does not contain other receptors linked to G proteins (G Protein–Coupled Receptor *(*GPCR)) similar to GHS-R, it is possible that UAG exerts influence by mechanisms independent from GPCR [16]. Currently, it is becoming clear that, in some cases, the biological effect of GHRL is mediated by the AG form with the existence of the UAG, while in others unacylated form may imitate AG activity, and even exert antagonistic effects [20,21,22]. In this sense, it is considered that the proportion AG/UAG is exceptionally important in reference to further research on the physiological and pathophysiological role of this hormone in the organism, e.g., in the scope of retaining glucose homeostasis or other regulatory processes, similar to UAG itself [16,20,21,22,23].

In all vertebrates, GHRL is produced mainly in the stomach, where the greatest intensity of mRNA translation processes, which encode hormone sequence, has been demonstrated [1,11,14,24]. It is released from cells of the organ, the so-called X/A-like cells, in humans also called P/D_1_ cells [1,11,14,25]. Research in recent years has found that GHRL is produced in the pancreas by endocrine and exocrine cells. It has been demonstrated that the pancreas is an important source of GHRL. It is currently known that GHRL is produced by the pancreatic islet cells, yet their type has remained slightly controversial so far: whether they are α-cells [26], β-cells [27], or a cell population currently being identified and defined as the “epsilon” (ε) islet cell type [28]. The ε cells are located in pancreatic islets in the same areas as β cells. They also develop from precursor cells, and the proteins Nkx2.2 and Pax4 affect their differentation. In the presence of Nkx2.2 and Pax4 pancreatic precursor cells differentiate into β-cells. In the absence these proteins precurosor cells do not differentiate to β-cells, but differentiation to ε cells is observed [29]. It was recently demonstrated that a loss of Pax6 protein results in a similar phenotype: a reduction of β cells number and, respectively, an expansion of ε cells [29]. Because Pax6 expression depends on Nkx2.2, it is probable that Nkx2.2, working above Pax6 on the same pathway, regulates differentiation of these cells [29,30]. However, research conducted in recent years indicates that Nkx2.2 is a necessary regulating agent for pancreas endocrine cell diversification [31]. The most recent research performed in rats indicates that exocrine pancreatic cells are also a source of GHRL. It has been demonstrated that expression of mRNA for GHRL and production of GHRL occurs in acinar cells of the pancreatic tumor cell line (Rat Pancreatic Acinar Tumor Cell Line AR42J), as well as in the normal human and rat exocrine pancreas [24,32,33]. GHRL mRNA expression was also revealed in other parts of the digestive tract, such as the gall bladder, esophagus, liver and spleen [24]. In addition, it has been found to be produced by other peripheral organs and systems (the kidney and urinary bladder; the respiratory system: the lung; the cardiovascular system: the heart and veins; the endocrine system: the thyroid and adrenal gland; the immune system: the lymphatic vessels, T and B lymphocytes, and neutrophils) [24]. Moreover, GHRL was identified in the central areas of the brain (arcuate nucleus and hypothalamus neurones) and in the pituitary gland [1,24]. It was also found in the skin, breast, buccal mucosa, muscle tissue, fat tissue and in neoplastic cell lines (the human medullary thyroid cancer cell line (NRK-49F), HL-1, ECC10, and MGN3-1) [24,34,35,36,37,38,39].

Caerulein-induced pancreatitis (CIP) is one of the best characterized and widely used experimental models of this disease [40]. In rats, hyperstimulation of the exocrine pancreas by cholecystokinin (CCK) or its analog, caerulein leads to the development of acute mild edematous pancreatitis. Caerulein is most commonly used. This model of acute pancreatitis is based on the theory of collocation of digestive and lysosomal enzymes in the course of this disease [41,42,43,44].

In physiological conditions, synthesis of pancreatic enzymes occurs in the endoplasmic reticulum. Newly synthesized proteins undergo modification in this reticulum including phosphorylation, sulfation and glucosylation. Then they are transported to the Golgi apparatus, and stocked in zymogene granularities. In physiological conditions, secretion of pancreatic digestive enzymes occurs by exocitosis. Exocitosis consists of movement of the secretory granules to the apical surface of acinar cells and secretion of enzymes to the lumen of pancreatic acini [45].

In the CIP model, intracellular digestive proenzymes activation occurs, induced by colocalization of zymogene granularities and lysosomal enzymes. As a result, there appear big vacuoles, whose acidic pH supports activation of trypsinogen by cathepsin B. Finally, it leads to autolysis of the pancreatic acinar cells with apical release of active digestive enzymes into the intraparenchymal space of the pancreas. Secondary periacinar and perilobular changes occur and inflammatory reaction develops in the pancreatic stroma. The changes develop quickly, reaching the maximum intensity between the third and sixth hour after caerulein infusion [43,46,47,48]. The histopathological examination confirms that there occurs a massive intraparenchymal edema associated with microcirculatory disorders and increased passage of proteins into the perivascular space. An infiltration of inflammatory cells and vacuolization of acinar cells also appears. In these processes are also involved enzymatic cascades activating hemostasis, the complement and kinin system [49,50,51,52,53,54,55].

The aim of this study was to investigate the effect of GHRL and caerulein on the functional ghrelin system in pancreatic acinar cells taking into account the role of sensory nerves.

## 2. Results

### 2.1. Influence of Ghrelin Administered Peripherally In Vivo on the GHS-R1a and GHRL Level of Gene Expression and Protein Production in the Pancreatic Acinar Cells with Intact and/or Deactivated Sensory Nerves in Rats in Basic Conditions and after Hyperstimulation with Caerulein in the In Vitro Model

#### 2.1.1. Determination of GHS-R1a Gene Expression and Protein Production

**Figure 1:**
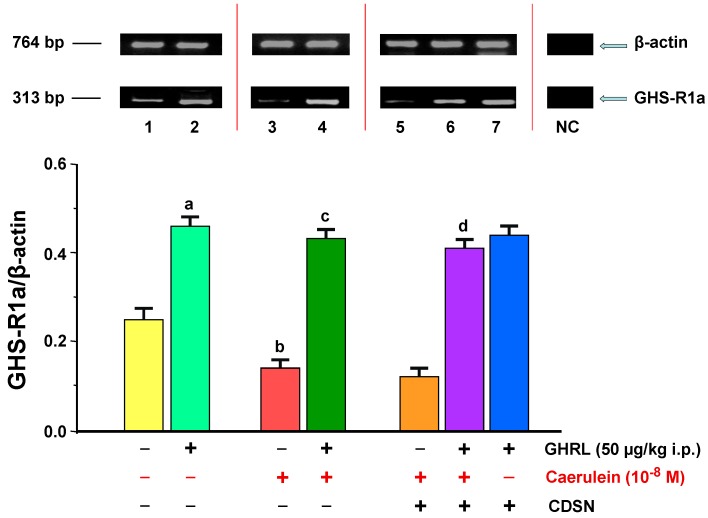
Analysis of the growth hormone secretagogue receptor 1a (GHS-R1a) gene expression determined by reverse transcriptase-polymerase chain reaction (RT-PCR) and densitometric analysis of GHS-R1A/*β-actin* mRNA ratio in pancreatic acinar cells: (line 1) acinar cells obtained from control sensory nerves (SN)-intact rats treated with saline, after isolation, acinar cells incubated in caerulein-free solution; (line 2) acinar cells obtained from SN-intact rats treated with ghrelin (GHRL), after isolation, acinar cells incubated in caerulein-free solution; (line 3) acinar cells obtained from SN-intact rats treated with saline, after isolation, acinar cells incubated in solution containing caerulein at a concentration of 10^−8^ M; (line 4) acinar cells obtained from SN-intact rats treated with GHRL, after isolation, acinar cells incubated in solution containing caerulein at a concentration of 10^−8^ M; (line 5) acinar cells obtained from rats with capsaicin deactivation of SN (CDSN) and treated with saline, after isolation, acinar cells incubated in solution containing caerulein at a concentration of 10^−8^ M; (line 6) acinar cells obtained from rats with CDSN and treated with GHRL, after isolation, acinar cells incubated in solution containing caerulein at a concentration of 10^−8^ M; (line 7) acinar cells obtained from rats with CDSN and treated with GHRL, after isolation, acinar cells incubated in caerulein-free solution. NC = negative control. Reference gene: β-actin. ^a,b^ *p* < 0.05 compared to control acinar cells obtained from rats with intact SN (line 1); ^c^ *p* < 0.05 compared to acinar cells stimulated with caerulein after isolation from SN-intact rats treated with saline (line 3); ^d^ *p* < 0.05 compared to acinar cells stimulated with caerulein after isolation from rats with CDSN and treated with saline (line 5). In each experimental group, the number of observations was at least 6.

The GHS-R1a mRNA signal in isolated pancreatic acinar cells under in vitro conditions was determined in all examined samples. In the animal control group (0.9% NaCl), the ratio of GHS-R1a/*β-actin* gene expression was 0.25 ± 0.01. Intraperitoneal (i.p.) administration of exogenous GHRL in rats in a fixed dose of 50.0 µg/kg, 48 h prior to the in vitro experiment, resulted in a statistically significant upregulation of the ratio of GHS-R1a/*β-actin* mRNA signal to the level of 0.46 ± 0.02 (Figure 1).

Hyperstimulation of pancreatic acinar cells with the selected concentration of caerulein (10^−8^ M), for 5 h, resulted in a statistically significant downregulation of the ratio of GHS-R1a/*β-actin* gene expression to the level of 0.14 ± 0.005 as compared to the rat control group (0.9% NaCl). Intraperitoneal administration of exogenous GHRL in vivo in a dose of 50.0 µg/kg, 48 h prior to the use of caerulein in vitro, resulted in a statistically significant upregulation of the ratio of GHS-R1a/*β-actin* mRNA signal to the value of 0.43 ± 0.02 (Figure 1).

CDSN, as compared to the group with caerulein and intact SN, had no influence on the ratio of GHS-R1a/*β-actin* mRNA signal in pancreatic acinar cells, stimulated with caerulein at a concentration of 10^−8^ M. The signal ratio was maintained at 0.12 ± 0.005. Peripheral administration of exogenous GHRL, in vivo, in a dose of 50.0 µg/kg i.p. in the animal group with CDSN, 48 h prior to the administration of the pancreatic secretagogue, with concentration of 10^−8^ M, in vitro, resulted in a statistically significant upregulation of the GHS-R1a/*β-actin* gene expression ratio to 0.41 ± 0.02 vs. the group without GHRL. This change caused an alignment of the examined parameter as compared to the group of rats receiving the same dose of GHRL, with subsequent administration of caerulein in the animal group with intact SN (0.43 ± 0.02). A comparison of the GHS-R1a/β-actin gene expression ratio in pancreatic acinar cells in animals receiving exogenous GHRL in vivo (50.0 µg/kg i.p., 48 h prior to the cell isolation) between the group with intact SN (0.46 ± 0.02) and the group with CDSN (0.44 ± 0.02), showed no significant difference between them (Figure 1).

**Figure 2:**
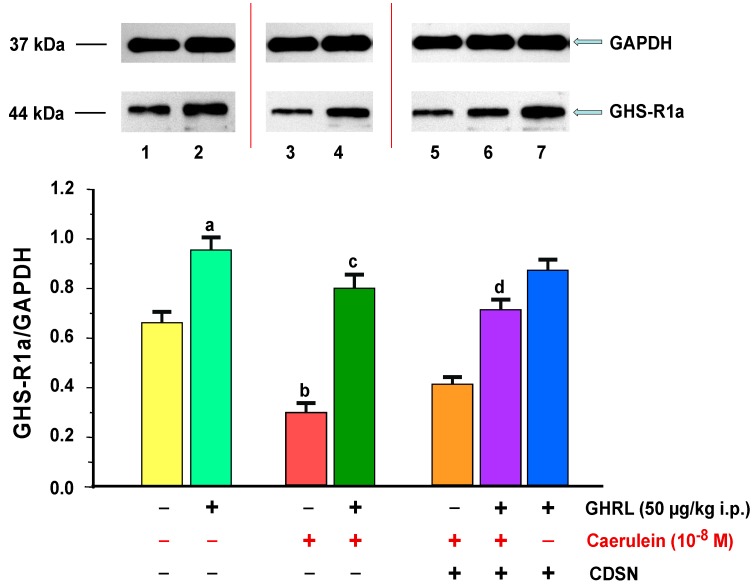
Analysis of the growth hormone secretagogue receptor 1a (GHS-R1a) protein production determined by the methods of immunoblotting and immunoprecipitation, and densitometric analysis of GHS-R1A/glyceraldehyde-3-phosphate dehydrogenase (GAPDH) protein ratio in pancreatic acinar cells: (line 1) acinar cells obtained from control sensory nerves (SN)-intact rats treated with saline, after isolation, acinar cells incubated in caerulein-free solution; (line 2) acinar cells obtained from SN-intact rats treated with ghrelin (GHRL), after isolation, acinar cells incubated in caerulein-free solution; (line 3) acinar cells obtained from SN-intact rats treated with saline, after isolation, acinar cells incubated in solution containing caerulein at a concentration of 10^−8^ M; (line 4) acinar cells obtained from SN-intact rats treated with GHRL, after isolation, acinar cells incubated in solution containing caerulein at a concentration of 10^−8^ M; (line 5) acinar cells obtained from rats with capsaicin deactivation of SN (CDSN) and treated with saline, after isolation, acinar cells incubated in solution containing caerulein at a concentration of 10^−8^ M; (line 6) acinar cells obtained from rats with CDSN and treated with GHRL, after isolation, acinar cells incubated in solution containing caerulein at a concentration of 10^−8^ M; (line 7) acinar cells obtained from rats with CDSN and treated with GHRL, after isolation, acinar cells incubated in caerulein-free solution. Reference protein: GAPDH. ^a,b^ *p* < 0.05 compared to control acinar cells obtained from rats with intact SN (line 1); ^c^ *p* < 0.05 compared to acinar cells stimulated with caerulein after isolation from SN-intact rats treated with saline (line 3); ^d^ *p* < 0.05 compared to acinar cells stimulated with caerulein after isolation from rats with CDSN and treated with saline (line 5). In each experimental group, the number of observations was at least 6.

In the isolated pancreatic acinar cells in vitro, in all animal groups, the presence of the GHS-R1a protein was shown. In the control conditions (0.9% NaCl), the ratio of GHS-R1a/GAPDH protein was 0.67 ± 0.03. Intraperitoneal administration of exogenous GHRL to rats in a dose of 50.0 µg/kg, 48 h prior to the in vitro experiment has led to a statistically significant upregulation of the GHS-R1a/GAPDH protein ratio to the level of 0.96 ± 0.04 (Figure 2).

Hyperstimulation of pancreatic acinar cells with caerulein at a concentration of 10^−8^ M for 5 h resulted in a statistically significant downregulation of the ratio of GHS-R1a/GAPDH protein production, the smallest downregulation, when compared to the other concentrations, which was 0.30 ± 0.01. The peripheral use of exogenous GHRL in vivo in a dose of 50.0 µg/kg i.p., 48 h prior to in vitro application of caerulein, resulted in a statistically significant upregulation of the ratio of GHS-R1a/GAPDH protein to the level of 0.80 ± 0.04 (Figure 2).

CDSN did not change the ratio of GHS-R1a/GAPDH protein in pancreatic acinar cells stimulated with caerulein at a concentration of 10^−8^ M. The ratio remained at the level of 0.42 ± 0.02 as compared to the group with secretagogue and intact SN. In the group of animals with CDSN, intraperitoneal administration of exogenous GHRL in vivo in a selected dose of 50.0 µg/kg, 48 h prior to the administration of the pancreatic secretagogue at a concentration of 10^−8^ M in vitro, caused a statistically significant increase in GHS-R1a/GAPDH protein production, which reached the level of 0.72 ± 0.03. This change caused an alignment of the ratio examined as compared to the group of animals receiving an identical dose of GHRL, with subsequent administration of caerulein with intact SN (0.80 ± 0.04). A comparison the ratio of GHS-R1a/GAPDH protein production in pancreatic acinar cells of rats receiving exogenous GHRL in vivo, in a fixed-dose of 50.0 µg/kg i.p., 48 h prior to the cell isolation between the group of rats with intact SN (0.96 ± 0.04), and the group with CDSN (0.88 ± 0.04), showed no significant differences between them (Figure 2).

#### 2.1.2. Determination of GHRL Gene Expression and Protein Production

**Figure 3:**
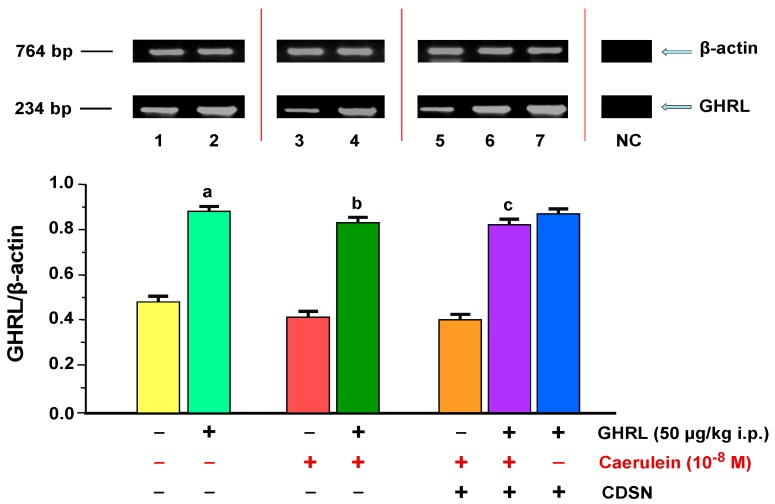
Analysis of ghrelin (GHRL) gene expression determined by reverse transcriptase-polymerase chain reaction (RT-PCR) and densitometric analysis of GHRL/*β-actin* mRNA ratio in pancreatic acinar cells: (line 1) acinar cells obtained from control sensory nerves (SN)-intact rats treated with saline, after isolation, acinar cells incubated in caerulein-free solution; (line 2) acinar cells obtained from SN-intact rats treated with GHRL, after isolation, acinar cells incubated in caerulein-free solution; (line 3) acinar cells obtained from SN-intact rats treated with saline, after isolation, acinar cells incubated in solution containing caerulein at a concentration of 10^−8^ M; (line 4) acinar cells obtained from SN-intact rats treated with GHRL, after isolation, acinar cells incubated in solution containing caerulein at a concentration of 10^−8^ M; (line 5) acinar cells obtained from rats with capsaicin deactivation of SN (CDSN) and treated with saline, after isolation, acinar cells incubated in solution containing caerulein at a concentration of 10^−8^ M; (line 6) acinar cells obtained from rats with CDSN and treated with GHRL, after isolation, acinar cells incubated in solution containing caerulein at a concentration of 10^−8^ M; (line 7) acinar cells obtained from rats with CDSN and treated with GHRL, after isolation, acinar cells incubated in caerulein-free solution. NC = negative control. Reference gene: β-actin. ^a^ *p* < 0.05 compared to control acinar cells obtained from rats with intact SN (line 1); ^b^ *p* < 0.05 compared to acinar cells stimulated with caerulein after isolation from SN-intact rats treated with saline (line 3); ^c^ *p* < 0.05 compared to acinar cells stimulated with caerulein after isolation from rats with CDSN and treated with saline (line 5). In each experimental group, the number of observations was at least 6.

GHRL gene expression was determined in isolated pancreatic acinar cells obtained in vitro from all animal groups examined. The ratio of GHRL/*β-actin* mRNA signal in the control group (0.9% NaCl) was 0.48 ± 0.02. Peripheral administration of exogenous GHRL to rats in a dose of 50.0 µg/kg i.p., 48 h prior to in vitro experiment, resulted in a statistically significant upregulation of the ratio of GHRL/*β-actin* gene expression to 0.88 ± 0.04 (Figure 3).

Caerulein hyperstimulation of pancreatic acinar cells with a selected concentration of secretagogue—10^−8^ M for 5 h did not, compared to the control group of rats (0.9% NaCl), result in a change the ratio of GHRL/*β-actin* gene expression, which was 0.41 ± 0.02. Intraperitoneal administration of exogenous GHRL in vivo, in a dose of 50.0 µg/kg, 48 h prior to the administration of caerulein in vitro, resulted in a statistically significant upregulation of the ratio of GHRL/*β-actin* mRNA signal to the level of 0.83 ± 0.04 (Figure 3).

CDSN, as compared to the group with caerulein and intact SN, had no affect on the ratio of GHRL/*β-actin* gene expression in pancreatic acinar cells stimulated with secretagogue at a concentration of 10^−8^ M. The ratio maintained the level of 0.40 ± 0.02. Intraperitoneal administration of exogenous GHRL in vivo to the animal group with CDSN in a dose of 50.0 µg/kg intraperitoneally (i.p.), 48 h prior to the administration of caerulein, at a concentration of 10^−8^ M in vitro, resulted in a statistically significant upregulation of the ratio of GHRL/*β-actin* gene expression to the level of 0.82 ± 0.04 vs. the group without GHRL. This upregulation caused an alignment of the ratio examined as compared to the group of rats receiving an identical dose of GHRL, with subsequent administration of secretagogue with intact SN (0.83 ± 0.04). A comparison the ratio of GHRL/*β-actin* mRNA signal in pancreatic acinar cells of animals receiving exogenous GHRL in vivo, in a fixed-dose of 50.0 µg/kg i.p., 48 h prior to the cell isolation, between the group of animals with intact SN (0.88 ± 0.04), and the group with CDSN (0.87 ± 0.04), showed no significant differences between them (Figure 3).

The presence of the GHRL protein in isolated pancreatic acinar cells in the in vitro conditions has been shown in all examined samples. In the analysis, a single group of bands was detected at the size level of 13 kDa in the cell extracts, which corresponds to a mature hormone, wherein the Ser-3 hydroxyl group is acylated with an *n-*octane acid; it is a 28-amino acid peptide, AG. No other group bands were observed.

**Figure 4:**
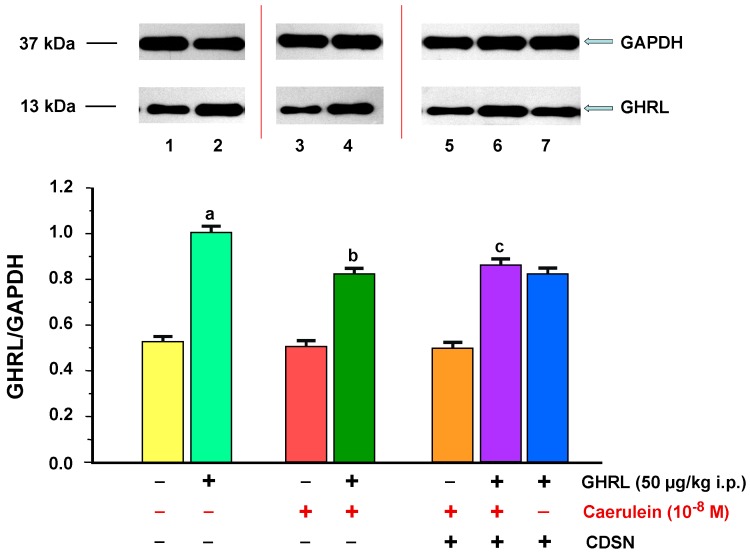
Analysis of ghrelin (GHRL) protein production determined by the methods of immunoblotting and immunoprecipitation, and densitometric analysis of GHRL/glyceraldehyde-3-phosphate dehydrogenase (GAPDH) protein ratio in pancreatic acinar cells: (line 1) acinar cells obtained from control sensory nerves (SN)-intact rats treated with saline, after isolation, acinar cells incubated in caerulein-free solution; (line 2) acinar cells obtained from SN-intact rats treated with GHRL, after isolation, acinar cells incubated in caerulein-free solution; (line 3) acinar cells obtained from SN-intact rats treated with saline, after isolation, acinar cells incubated in solution containing caerulein at a concentration of 10^−8^ M; (line 4) acinar cells obtained from SN-intact rats treated with GHRL, after isolation, acinar cells incubated in solution containing caerulein at a concentration of 10^−8^ M; (line 5) acinar cells obtained from rats with capsaicin deactivation of SN (CDSN) and treated with saline, after isolation, acinar cells incubated in solution containing caerulein at a concentration of 10^−8^ M; (line 6) acinar cells obtained from rats with CDSN and treated with GHRL, after isolation, acinar cells incubated in solution containing caerulein at a concentration of 10^−8^ M; (line 7) acinar cells obtained from rats with CDSN and treated with GHRL, after isolation, acinar cells incubated in caerulein-free solution. Reference protein: GAPDH. ^a^ *p* < 0.05 compared to control acinar cells obtained from rats with intact SN and treated with saline (line 1); ^b^ *p* < 0.05 compared to acinar cells stimulated with caerulein after isolation from SN-intact rats treated with saline (line 3); ^c^ *p* < 0.05 compared to acinar cells stimulated with caerulein after isolation from rats with CDSN and treated with saline (line 5). In each experimental group, the number of observations was at least 6.

The ratio of GHRL/GAPDH protein in the animal control group (0.9% NaCl) was 0.53 ± 0.02. Intraperitoneal administration of exogenous GHRL to animals in a dose of 50.0 µg/kg, 48 h before the experiment in vitro, resulted in a statistically significant upregulation of the ratio of GHRL/GAPDH protein production to the level of 1.02 ± 0.04 (Figure 4).

Stimulation of pancreatic acinar cells with the usage of a selected concentration of caerulein of 10^−8^ M for 5 h caused a statistically insignificant downregulation tendency of the ratio of GHRL/GAPDH protein to the level of 0.51 ± 0.02, compared to the control rat group (0.9% NaCl). Administration of exogenous GHRL to animals in vivo in a dose of 50.0 µg/kg i.p., 48 h prior to the use of the pancreatic secretagogue in vitro, resulted in a statistically significant upregulation of the ratio of GHRL/GAPDH protein production, to 0.83 ± 0.03 (Figure 4).

CDSN did not influence the level of the ratio of GHRL/GAPDH protein in the pancreatic acinar cells stimulated with caerulein at a concentration of 10^−8^ M (0.50 ± 0.02), compared with the group with caerulein and intact SN. Peripheral administration of exogenous GHRL in vivo in a dose of 50.0 µg/kg i.p. 48 h before the administration of caerulein (10^−8^ M) in vitro, in the animal group with CDSN, resulted in a statistically significant upregulation of the ratio of GHRL/GAPDH protein to the value of 0.87 ± 0.03 vs. the group without GHRL. This upregulation caused an alignment of the ratio examined as compared to the group of rats receiving the same dose of GHRL, with subsequent administration of caerulein with intact SN (0.83 ± 0.03). A comparison of the ratio of GHRL/GAPDH protein production in pancreatic acinar cells of rats receiving exogenous GHRL in vivo, in a fixed-dose of 50.0 µg/kg i.p., 48 h prior to the cell isolation, between the group of rats with intact SN (1.02 ± 0.04), and the group with CDSN (0.83 ± 0.03), showed no significant differences between them (Figure 4).

### 2.2. Influence of Ghrelin on the GHS-R1a and GHRL Level of Gene Expression and Protein Production in the AR42J Cells in Basic Conditions and after Hyperstimulation with Caerulein In Vitro

#### 2.2.1. Determination of GHS-R1a Gene Expression and Protein Production

**Figure 5:**
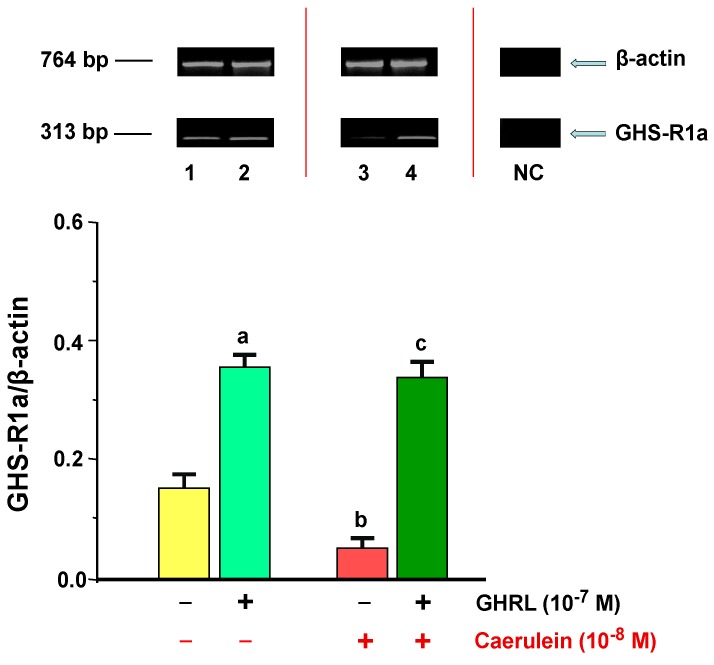
Analysis of the growth hormone secretagogue receptor 1a (GHS-R1a) gene expression determined by reverse transcriptase-polymerase chain reaction (RT-PCR) and densitometric analysis of GHS-R1A/*β-actin* mRNA ratio in AR42J cells: (line 1) control cells incubated in caerulein-and GHRL-free solution; (line 2) cells incubated in caerulein-free solution containing GHRL at a concentration of 10^−7^ M; (line 3) cells incubated in solution containing caerulein at a concentration of 10^−8^ M without addition of GHRL; (line 4) cells incubated in solution containing caerulein at a concentration of 10^−8^ M and GHRL at a concentration of 10^−7^ M. NC = negative control. Reference gene: β-actin. ^a,b^ *p* < 0.05 compared to control cells incubated in caerulein-and GHRL-free solution (line 1); ^c^ *p* < 0.05 compared cells incubated in solution containing caerulein without addition of GHRL (line 3). In each experimental group, the number of observations was at least 6.

The gene expression of GHS-R1a type in AR42J cells, was determined in all examined samples. In the control group, the ratio of GHS-R1a/*β-actin* mRNA signal was 0.17 ± 0.01. An addition of exogenous GHRL (10^−7^ M) resulted in a statistically significant upregulation of the ratio of GHS-R1a/*β-actin* gene expression to the level of 0.36 ± 0.02 (Figure 5).

Hyperstimulation of AR42J cells with the selected concentration of caerulein—10^−8^ M, resulted in a statistically significant downregulation of the ratio of GHS-R1a/*β-actin* mRNA signal to the level of 0.05 ± 0.003 after 48 h of incubation as compared to the control group. Incubation of the cell cultures with combination of GHRL (10^−7^ M), and caerulein (10^−8^ M), resulted in a statistically significant upregulation of the ratio of GHS-R1a/*β-actin* gene expression to the value of 0.34 ± 0.02 after 48 h of incubation (Figure 5).

**Figure 6:**
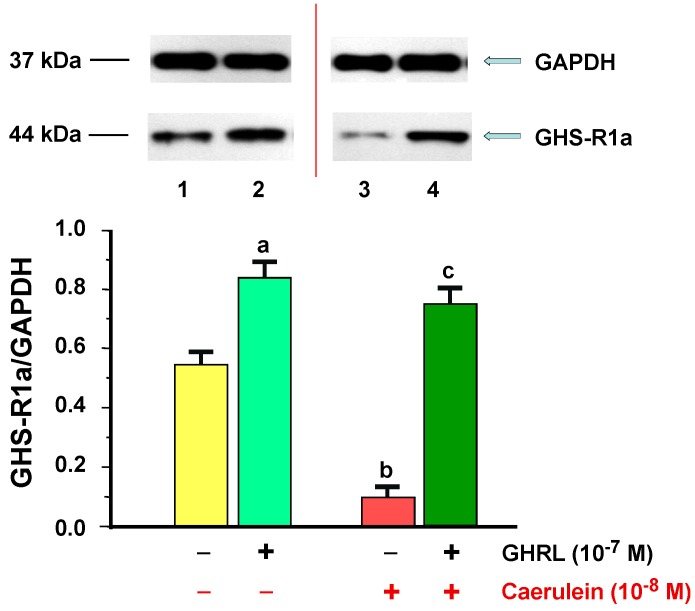
Analysis of the growth hormone secretagogue receptor 1a (GHS-R1a) protein production determined by the methods of immunoblotting and immunoprecipitation, and densitometric analysis of GHS-R1A/glyceraldehyde-3-phosphate dehydrogenase (GAPDH) protein ratio in AR42J cells: (line 1) control cells incubated in caerulein-and GHRL-free solution; (line 2) cells incubated in caerulein-free solution containing GHRL at a concentration of 10^−7^ M; (line 3) cells incubated in solution containing caerulein at a concentration of 10^−8^ M without addition of GHRL; (line 4) cells incubated in solution containing caerulein at a concentration of 10^−8^ M and GHRL at a concentration of 10^−7^ M. Reference protein: GAPDH. ^a,b^ *p* < 0.05 compared to control cells incubated in caerulein-and GHRL-free solution (line 1); ^c^ *p* < 0.05 compared cells incubated in solution containing caerulein without addition of GHRL (line 3). In each experimental group, the number of observations was at least 6.

The amount of GHS-R1a type proteins in the AR42J cells was determined in all examined samples. The ratio of GHS-R1a/GAPDH protein level in the control cells was 0.55 ± 0.02 and significantly increased in the cells treated with GHRL (10^−7^ M). The ratio of GHS-R1a/GAPDH in control was 0.84 ± 0.03 after 48 h of incubation (Figure 6).

Addition of caerulein (10^−8^ M) to the AR42J cell cultures significantly downregulated the protein expression of GHS-R1a, as compared to the control group. The ratio of GHS-R1a/GAPDH was 0.30 ± 0.01 after 48 h of incubation. Exposition of the AR42J cells to the combination of GHRL (10^−7^ M) and caerulein (10^−8^ M) resulted in a significant upregulation of signal for GHS-R1a after 48 h of incubation as compared to the caerulein alone treated AR42J cell culture; the ratio was 0.75 ± 0.03 (Figure 6).

#### 2.2.2. Determination of GHRL Gene Expression and Protein Production

**Figure 7:**
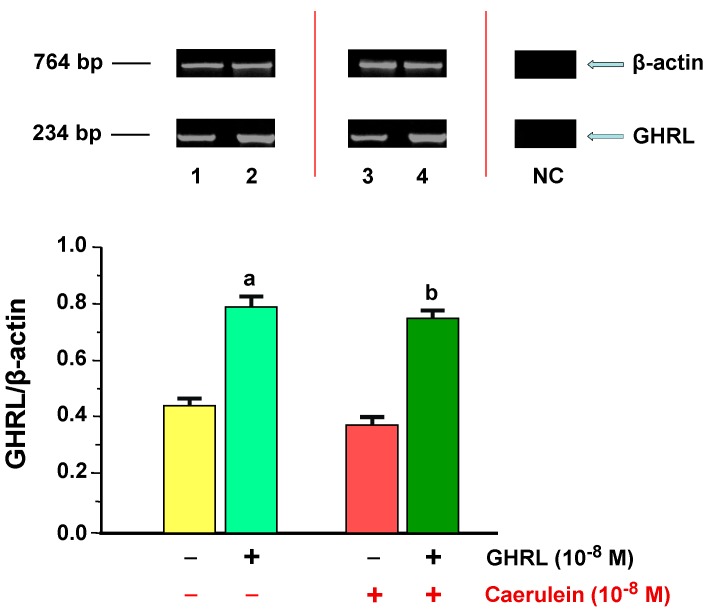
Analysis of ghrelin (GHRL) gene expression determined by reverse transcriptase-polymerase chain reaction (RT-PCR) and densitometric analysis of GHS-R1A/*β-actin* mRNA ratio in AR42J cells: (line 1) control cells incubated in caerulein-and GHRL-free solution; (line 2) cells incubated in caerulein-free solution containing GHRL at a concentration of 10^−7^ M; (line 3) cells incubated in solution containing caerulein at a concentration of 10^−8^ M without addition of GHRL; (line 4) cells incubated in solution containing caerulein at a concentration of 10^−8^ M and GHRL at a concentration of 10^−7^ M. NC= negative control. Reference gene: β-actin. ^a^ *p* < 0.05 compared to control cells incubated in caerulein-and GHRL-free solution (line 1); ^b^ *p* < 0.05 compared cells incubated in solution containing caerulein without addition of GHRL (line 3). In each experimental group, the number of observations was at least 6.

The GHRL mRNA signal in AR42J cells, was determined in all examined samples. In the control the ratio of GHRL/*β-actin* gene expression was 0.43 ± 0.02. Addition of exogenous GHRL (10^−7^ M) resulted in a statistically significant upregulation of the ratio of GHRL/*β-actin* to the level of 0.79 ± 0.03 after 48 h of incubation (Figure 7).

Hyperstimulation of AR42J cells with a selected concentration of secretagogue of 10^−8^ M, resulted in a statistically significant downregulation of the ratio of GHRL/*β-actin* mRNA signal to the level of 0.37 ± 0.02 after 48 h of incubation as compared to the control group. Incubation of the cell cultures with combination of GHRL (10^−7^ M), and caerulein (10^−8^ M), resulted in a statistically significant upregulation of the ratio of GHRL/β-actin gene expression to the value of 0.75 ± 0.03 after 48 h of incubation (Figure 7).

**Figure 8:**
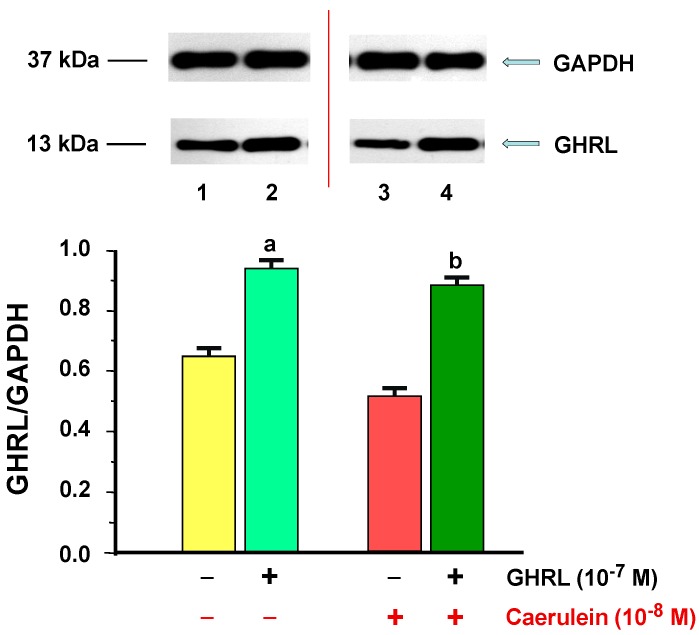
Analysis of ghrelin (GHRL) protein production determined by the methods of immunoblotting and immunoprecipitation, and densitometric analysis of GHS-R1A/glyceraldehyde-3-phosphate dehydrogenase (GAPDH) protein ratio in AR42J cells: (line 1) control cells incubated in caerulein-and GHRL-free solution; (line 2) cells incubated in caerulein-free solution containing GHRL at a concentration of 10^−7^ M; (line 3) cells incubated in solution containing caerulein at a concentration of 10^−8^ M without addition of GHRL; (line 4) cells incubated in solution containing caerulein at a concentration of 10^−8^ M and GHRL at a concentration of 10^−7^ M. Reference protein: GAPDH. ^a^ *p* < 0.05 compared to control cells incubated in caerulein-and GHRL-free solution (line 1); ^b^ *p* < 0.05 compared cells incubated in solution containing caerulein without addition of GHRL (line 3). In each experimental group, the number of observations was at least 6.

GHRL protein was detected in all examined samples isolated from exocrine cells line AR42J. In the analysis, a single group of bands was detected at approximately 13 kDa in the extract from AR42J cells and it corresponds to the *n-*octanoylated mature 28-amino-acid GHRL peptide. No other bands were observed. The ratio of GHRL/GAPDH protein expression in the control group was 0.65 ± 0.02 and significantly increased in the cells treated by GHRL (10^−7^ M) after 48 h of incubation; the ratio was 0.94 ± 0.03 (Figure 8).

Application of caerulein (10^−8^ M) to the AR42J cells did not significantly downregulate GHRL protein level, as compared to the control group with ratio of GHRL/GAPDH 0.52 ± 0.02 after 48 h of incubation. Incubation of the cell cultures with combination of GHRL (10^−7^ M), and caerulein (10^−8^ M) significantly increased the GHRL protein level, as compared to the value obtained from caerulein alone treated cells. The ratio of GHRL/GAPDH in these cells was 0.89 ± 0.03 after 48 h of incubation (Figure 8).

## 3. Discussion

In our present study, we demonstrated that exogenous ghrelin (GHRL) and caerulein regulate the functional ghrelin system in pancreatic acinar cells and that this phenomenon occurs partly with the sensory nerves (SN) involvement. To our knowledge, this is the first report indicating in both in vivo and in vitro studies, that this polypeptide and SN are involved in the regulation of GHS-R1a and GHRL expression in the pancreatic acinar cells.

The results of our study demonstrated that the GHS-R1a type and the acylated GHRL gene and protein are expressed in pancreatic acinar cells obtained from rats with intact SN and pancreatic AR42J cells, and above effects are in agreement with the reports of Lai et al. [32,33].

Human GHS-R has been identified in chromosome 3, on position q26–27 [2]. It belongs to the family of GPCR receptors and is a typical metabotropic receptor (7-transmembrane (7TM), transmembrane). *GHS-R* gene consists of two exons; the first one encodes TM1–TM5, and the second encodes TM6–TM7. The GHS-R gene promotor region does not contain typical amino acid sequences (TATA, CAAT, and GC), but possesses numerous binding sites for transcription factors and hormones, inter alia, estrogens [56].

GHR-R is expressed as two separate mRNAs [57]. The first one, GHS-R type 1a, encodes 7TM GPCR with a binding and possesses functional properties in accordance with the role of the ghrelin receptor. It is built of 366 amino acids and its molecule mass equals 41 kDa [58]. The other one, mRNA GHS-R type 1b, produced as a result of alternative splicing, is built of 289 amino acids and possesses only five domains. Type b is the product of the first exon only and encodes only five out of the seven predicted domains. Hence, this type is the form shortened by the COOH-terminal of type 1a receptor, and it does not possess transmembrane domains 6 and 7, which makes it physiologically inactive. However, a growing number of reports argue for type b receptor to also have a specific biological function [57].

GHS-R has a few homologues, the endogenous ligands of which are the digestive tract peptides or neuropeptides [59]. This super-family contains receptors for GHRL, motilin (MTL), neuromedin U (NmU) [60] and neurotensin [61]. All of them have been identified in the digestive tract organs and are responsible for regulation of its motility and other functions. GHS-R has the highest homology with the MTL receptor (MTLR); the human form has 52% homology as far as amino acid composition is concerned [62].

Apart from that, their ligands—GHRL and MTL peptides—have similar amino acid sequences. Research demonstrated that MTL has a very low ability to activate GHS-R, unlike GHRL, which cannot activate MTLR [63].

GHS-R appears highly conserved in all studied vertebrate species, including many mammals, chicken and a species of fish from the family of pufferfish (blowfish)—*Tetraodontidae* (Fugu), and it is characterized by a very similar amino acid sequence [64,65]. This narrow conservation indicates that this receptor fulfills vital physiological functions.

It is suggested that there also exist other innovative, so far unidentified, sub-types of this receptor. This is because, during examination of the binding of isotope-marked GHRL to a line of fat tissue cells 3T3-L1, the attempt to identify the receptor type to which that hormone bound proved to be unsuccessful [66]. Moreover, both GHRL and the UAG forms bind with in vitro grown H9c2 cardiomyocytes, which do not show GHS-R expression [67,68]. It is also suspected that UAG activity occurs with a receptor other than GHS-R type 1a as a mediator [61]. Hence, it is necessary to continue subsequent research, paying special attention to the unidentified type of GHS-R.

The presence of mRNA signal for GHS-R and/or its production has been demonstrated in an organism both in the central structures [57,69,70] and in the peripheral tissues [24,70,71,72]. Genetic expression of GHS-R type 1a has been identified in the thyroid, pancreas, spleen, cardiac muscle and adrenal gland. On the other hand, GHS-R type 1b has been widely detected in the following systems: digestive, respiratory, cardiovascular, endocrine, reproductive, excretory and immune. Moreover, it has also occurred in the skin, breast, buccal mucosa, muscle and fat tissue [24,70,71,72].

Research conducted in recent years has demonstrated the presence of GHS-R in the endocrine and exocrine pancreatic cells, in the endocrine pancreatic tumor and in the pancreatic acinar tumor cell line, AR42J [24,27,28,32,33]. For the first time, a weak mRNA GHS-R signal was detected in the rat pancreas during hybridization in situ [63]. Indeed, not only transcripts for GHRL, but also for GHS-R have been detected in pancreatic tissue, both in humans [24,27,73] and in rats [11,26]. Immunohistochemical research conducted on rat pancreatic tissue revealed the presence of this receptor in the majority of α cells and in some but not all β cells [74]. It has recently been confirmed in human pancreatic islets [22], supporting the idea of autocrine/paracrine response of both cell types—α and β—to GHRL. In addition, mRNA signal and GHS-R type 1a production was observed in acinar cells and in the human and rat pancreas. Expression of mRNA for GHS-R type 1a and production of protein for this receptor was also found in AR42J cell line [24,32,33].

A wide distribution of GHS-R indicates that the receptor fulfills multiple physiological functions in an organism [75].

The research on the mechanisms of GH expression stimulation by substances led to the discovery of individual receptors. One of the stimulation pathways is the influence on GHRH-R by GHRH, whose second messenger is 3′–5′-cyclic adenosine monophosphorate (cAMP), then activates protein kinase A. This means that GHS-R is connected to sub-unit G_s_ [1].

However, unlike this pathway, GHRL works through the individual GHS-R, activating phospholipase C, generates inositol 1,4,5-triphosphate (IP_3_) and 1,2-diacyloglicerol (DAG), thus leading to an increase in intracellular Ca^2+^ ions, which indicates the fact that GHS-R is linked to G_q_. sub-unit [1]. GHS-R activation also leads to repression of potassium (K^+^) channels, which enables Ca^2+^ ions to come through voltage-gated L-type channels, not T-type channels. Synthetic GHS, GHRL and des-Gln^14^ bind with high affinity to GHS-R type 1a.

We have shown that in vivo or in vitro administration of exogenous GHRL in rats with intact SN or in AR42J cells culture resulted in a statistically significant upregulation of both GHS-R1a and AG gene expression and protein levels. However, application of caerulein caused a significant downregulation of GHS-R1a. Lai et al. [33] have reported that GHS-R mRNA and protein levels were downregulated by AP and upregulated by gastric acid inhibition, whereas they remained unchanged after food deprivation. In contrast, GHRL expression did not exhibit any significant changes in these conditions.

We have shown that exposure of the pancreatic acini cells obtained from rats with intact SN and acinar AR42J cells to the combination of GHRL and caerulein resulted in a statistically significant upregulation of both; GHS-R1a type and the GHRL protein and gene expression in the cells as compared to the caerulein alone treated pancreatic acinar cells and cultures. Our previous study [76], concerning the pancreatoprotective effect of GHRL in AP, has shown that increasing concentration of this peptide given intraperitoneally or intracerebroventriculary, resulted in dose-dependent rise of plasma GHRL concentration. The reduction of this peptide occurs in the course of caerulein-induced pancreatitis (CIP). GHRL given prior to the CIP protected the pancreas against the damage caused by AP. This was accompanied by large increases of GHRL plasma concentration. It is likely that the above rises of blood GHRL level are related to the increased production of this peptide and its receptor in the pancreatic acinar cells. Previous research studies, both in humans [77,78,79,80] and animals [81], indicated that GHRL could be used as a prognostic factor in AP and could be recognized as a good marker of the severity of this disease.

The results, published by us [76,82] and other researchers [83,84,85,86,87,88,89], clearly indicate that GHRL effectively protects the pancreas against AP induced by caerulein, sodium taurocholate or ischemia/reperfusion (I/R). Dembinski et al. [83] have reported that administration of GHRL attenuates pancreatic damage in CIP. This protective effect seems to be related to the inhibition in inflammatory process and the reduction in liberation of pro-inflammatory interleukin (IL)-1beta (β). Dembinski et al. [84] and Ceranowicz et al. [85] also demonstrated that administration of this peptide inhibits the development of I/R-induced pancreatitis and CIP, and this effect is mediated by its influence on the release of growth hormone (GH) and insulin-like growth factor (IGF)-1. Warzecha et al. [86] showed that treatment with GHRL exhibits therapeutic effect in CIP, which is related, at least in part, to the improvement of pancreatic blood flow (PBF), reduction in IL-1 β and stimulation of pancreatic cell proliferation. Bukowczan et al. [87] have reported that GHRL exerts a pronounced therapeutic effect against I/R-induced pancreatitis. The mechanisms involved are likely multifactorial and are mediated by its anti-inflammatory, as well as anti-oxidative properties. Zhou et al. [88] showed that this polypeptide inhibits the development of AP induced by sodium taurocholate. It exerts the therapeutic effects through inhibiting NF-kappaB (NF-κB) expression, thereby blocking the inflammatory signal transduction pathway and reducing the release of inflammatory media and cytokines. These researchers also showed that GHRL attenuates the severity of acute lung injury induced by AP. The reduction of neutrophil sequestration, limitation of proinflammatory cytokines release, and inhibition of pulmonary substance P (SP) expression may be the mechanisms involved in the therapeutic effect of this peptide [89].

Our studies have shown that CDSN maintained the adverse effect of caerulein hyperstimulation on GHS-R1a, causing its statistically significant reduction in the pancreatic acinar cells. GHRL peripheral administration in vivo reversed this adverse effect on the receptor. CDSN did not affect the signals for the GHRL in the pancreatic acini, hyperstimulated with pancreatic secretagogue. The peripheral application of exogenous GHRL in vivo prior to the administration of caerulein in vitro, resulted in a statistically significant increase of the tested signals.

Our research showed that CDSN increases the weight of the pancreas and enlarges inflammatory changes in histopathology. Other tested characteristics, parameters and markers in the course of the CIP, exacerbated the disease course [76]. These observations are consistent with the data previously published [90,91,92,93,94,95].

Thin sensory fibers, primary SN, are a particular group of nerves, both functional and histological. They occur in the spinal nerves, where their number is 80% [96,97,98,99]. They have sensory function of and participate in local reflex response [98,99]. SN represent the population of thin fibers with varicose, at their ends releasing peptidergic mediators—neuropeptides, such as tachykinin [100,101] or calcitonin gene related peptide (CGRP) [102].

SN cells are distinguished by the presence of integral transmembrane subtype 1 vanilloid receptors from the group of transient receptors potential vanilloid type-1 (TRPV1). Their natural ligand is capsaicin—an organic compound from the group of alkaloids. It demonstrates the ability for a specific and strong local release of neuropeptides from sensory nerve endings, and, therefore, for an induction of their activity and neurogenic inflammation. Capsaicin is a selective neurotoxin for SN [96,103,104,105,106].

Previous studies have shown that the effect of stimulation of SN in the course of AP is dependent on the phase of inflammation. SN activation or administration of CGRP before AP induced with caerulein or I/R relieves pancreatic damage during the inflammatory response [92,93,94,107,108,109]. SN activation or administration of CGRP after the induction of inflammation causes exacerbation of the inflammatory response, and leads to functional failure, typical for the chronic pancreatitis [110,111], in the course of which there has been an increase of TRPV1 [112]. The research conducted by our team has also shown that ablation of sensory fibers enhances the inflammatory response and completely abolishes the protective effect of central leptin [91,113], LPS [90], or L-tryptophan [114] in the course of CIP.

It must therefore be assumed that if exogenous GHRL eliminates the negative impact of caerulein and CDSN on pancreatic acinar cells, causing an increase in the production of GHS-R1a and GHRL, then this mechanism can strongly participate in the pancreatoprotective activity of this polypeptide in the course of AP.

The presence of GHRL and its receptor in the pancreatic endocrine islet cells [27,28], exocrine acinar and AR42J cells [32,33], clearly show that this peptide is likely to play a physiological role in the modulation of pancreatic endocrine and exocrine function. However, the effect of GHRL on insulin secretion is still uncertain [26,115,116,117]. Action of GHRL on amylase secretion is also unclear [82,118,119,120,121]. GHRL was demonstrated to inhibit pancreatic exocrine secretion in anesthetized rats and amylase release from pancreatic lobules probably via stimulation of intra-pancreatic neurons. However, this substance failed to affect basal and/or CCK-stimulated secretion of this enzyme in vitro [118]. In other studies, intracerebroventricular administration of this peptide stimulated the pancreatic secretory function in conscious rats [119]. Intra-duodenal infusion of GHRL dose-dependently enhanced basal and stimulated amylase secretion, what was accompanied by an increase in plasma CCK concentration [82,120,121]. Lai et al. [32] have demonstrated the presence of GHRL and GHSR in AR42J cells, with subsequent activation of calcium signaling by binding of GHRL to its receptor. This observation, in conjunction with the information that GHRL regulates insulin release [21,122], prompted authors to hypothesize that the functional GHRL system exists in the exocrine pancreas. Moreover the ability of pancreatic endocrine and exocrine cells to produce GHRL and localization of its receptors in the acinar cells and in AR42J cells indicates that this peptide could play a certain role in the regulation of enzyme secretion via an auto-/paracrine fashion [32]. Simeone et al. [123] have shown that CCK-8 in a dose-dependent way increased intracellular Ca^2+^ concentration ([Ca^2+^]_i_) in AR42J cells and this could be presumably attributed to the intracellular mobilization of these ions [124]. Similarly, GHRL dose-dependently increases [Ca^2+^]_i_ in these cells [32]. Lai et al. [32,33] assumed that upregulation of GHSR expression may have a potential GHRL inhibitory action on pancreatic exocrine function.

Studies carried out by Lai et al. [32] clearly indicate the presence of GHRL system in the pancreas and its potential relation to the calcium signaling pathway in the modulation of pancreatic exocrine function. This information is biologically significant particularly in view of the fact that CCK-8, being a very important regulator of pancreatic enzyme secretion, triggers calcium signaling in these cells in a different fashion than GHRL. These results support the notion that the effect of GHRL on the increase of the [Ca^2+^]_i_ in AR42J cells was mediated via GHS-R.

Increasing literature data suggest that GHRL could play an important role in the physiology and pathophysiology of the pancreas.

In conclusion, GHRL stimulated its own expression and regulates the functional ghrelin system in pancreatic acinar cell in the course of the caerulein-induced damage. This mechanism seems to participate in the pancreatoprotective effect of its action in the course of AP.

## 4. Material and Methods

### 4.1. Reagents

GHRL was purchased from Bachem AG, Budendorf, Switzerland. Caerulein (Takus) was purchased from Pharmacia, GmbH, Erlangen, Germany. Capsaicin was purchased from Fluka, Buchs, Switzerland.

### 4.2. Experimental Protocol

The experimental protocol was divided into two general parts: in vivo and in vitro experiments.

#### 4.2.1. In Vivo Experiments

The experiments were carried out on Wistar male rats weighing 170.0–200.0 g. Animals were housed in cages in standard conditions at room temperature with a normal circadian rhythm; 12-h day/night cycle. Rats were deprived of food 24 h prior to the start of experiment, while drinking water was available ad libitum.

All experimental procedures performed in this study were approved by the Jagiellonian University Ethical Committee on Animals Experimentation (Permit No ZI/UJ/118/2001 released on 20 July 2001).

##### Experimental Protocol and Groups

Forty eight h before the procedure of pancreatic acinar cell isolation, GHRL at a dose of 50.0 µg/kg (dissolved in 0.5 mL of 0.9% NaCl) was administered intraperitoneally (i.p.) to animals with intact sensory nerves (SN) or with capsaicin deactivation of SN (CDSN). The control group received i.p. 0.5 mL of saline at the same time before isolation of pancreatic acinar cells. Seven days prior to study, CDSN was performed by capsaicin applied subcutaneously (s.c.) at a total dose of 100.0 mg/kg over 3 days as described previously in detail [90]. Animals without induction of CDSN received s.c. saline. Each experimental group consists 6-8 animals.

#### 4.2.2. In Vitro Experiments

##### Rat Pancreatic Acinar Cells Experimental Protocol and Groups

Pancreatic acinar cells were isolated by collagenase digestion of pancreases [125] obtained from the animals with intact SN or CDSN. As described above, 48 h earlier, during in vivo study, rats received i.p. saline or GHRL. After isolation, pancreatic acinar cells were incubated in caerulein-free or caerulein containing solution [126,127]. Caerulein concentration was 10^−8^ M, incubation time was 5 h.

##### AR42J Cells Experimental Protocol and Groups

The study was performed on Rat Pancreatic Acinar Tumor Cell Line—AR42J cells (American Type Culture Collection, Rockville, MD, USA). Cell culture was grown in the RPMI 1640 Medium supplemented with Glutamax-I (Gibco BRL, Gaithersburg, MD, USA) and 10% fetal bovine serum (FBS, heat-inactivated; Gibco-BRL, Grand Island, NY, USA) with addition of 100 U/mL penicillin and 100 µg/mL streptomycine (Sigma-Aldrich, St. Louis, MO, USA) in the standard conditions: 37 °C and 5% CO_2_ [127,128,129,130].

Twenty-four hours before the experiments, culture medium was replenished with fresh RPMI 1640 containing 2% FBS and without antibiotics. Cells cultures were plated at the initial density of 2 × 10^6^/mL in a 100 mm culture plate (Falcon 3047; Becton Dickinson, Lincoln Park, NJ, USA) and allowed to attach for 12 h. AR42J cells were incubated in GHRL-free or GHRL containing medium and in the presence or absence of caerulein or in a combination of the above. GHRL was given at a concentration of 10^−7^ M, caerulein at a concentration of 10^−8^ M, incubation time was 48 h. All experiments were repeated at least six times.

### 4.3. Reverse Transcriptase-Polymerase Chain Reaction (RT-PCR)

Total cellular RNA was isolated from the pancreatic acinar cells and AR42J cell culture using TRIzol Reagent (Gibco-BRL, Life Technologies, Gaithersburg, MD, USA) according to the manufacturer’s protocol [131]. Following the precipitation RNA was suspended in RNase-free water and its concentration has been estimated by measurement of absorbance at 260 nm wavelength. A260/A280 ratio has been calculated to establish the purity of isolates.

The integrity of the isolates was confirmed by 1% agarose-formaldehyde gel electrophoresis and ethidium bromide staining. Aliquoted RNA samples were stored at −80 °C until analysis. The first strand cDNA synthesis was performed employing the Reverse Transcription System (Promega Corp., Madison, WI, USA) using 1 µg of RNA. For polymerase chain reaction, 2 µL of cDNA and oligo primers were used. All PCR reactions were performed with application of Promega PCR reagents. Specific primers, as listed below, were synthesized by Sigma-Genosys (Pampisford, UK). Products of RT-PCR reactions were analyzed using EtBr agarose gel electrophoresis. The post PCR abundance of cDNA product for each sample was estimated employing Foto/Analyst Fotodyne System (Fotodyne Inc., Hartland, WI, USA) on ethidium bromide stained 2% agarose gel. Location of the predicted PCR products was confirmed using O’Gene Ruler 50 bp DNA Ladder (Fermentas GmbH, St. Leon-Rot, Germany). Results of the semi-quantitative analysis were expressed as ratio using *β-actin* gene product as a reference for each sample. The gene, primers sequences product length, characteristic annealing temperatures and references were summarized in Table 1. In each analysis of the PCR reaction, negative and positive controls were added. The PCR reaction has been performed without addition of cDNA template or with cDNA synthetized on human RNA template to confirm specificity of reaction.

### 4.4. Co-Immunoprecipitation

The protein extracts from the rat pancreatic acinar cells and AR42J cells were prepared as described elsewhere [133]. Samples containing 5–10 μg of proteins were incubated for 4 h at 4 °C on shaking platform with 5 μL of primary antibodies. Five micrograms of A-agarose were added to each sample and samples were incubated overnight at 4 °C. Complexes were washed three times with radio immune precipitation buffer. Then, 10 μL of Western blot sample buffer were added to each pellet, boiled for 5 min at 95 °C and loaded on the 10% or 12% SDS-polyacrylamide gel, transferred and subjected to the regular Western blot procedure.

#### Immunoblotting

Separated samples were transferred onto the PVDF membrane (BioRad, Hercules, CA, USA). Following transfer membrane was blocked with blocking buffer (5% non-fat dried milk in PBS) for 2 h at room temperature. Immobilized on the membrane protein samples were exposed for 1h to the primary antibody diluted 1:1000. Each membrane was washed three times for 15 min in TBST buffer (0.1 M Tris pH 8.0; 1.5 M NaCl; 0.5% TritonX-100). Secondary antibody diluted 1:5000 in blocking buffer was applied for 1h at room temperature. All antibodies and other chemical (primary (mouse monoclonal IgG_1_ anti GAPDH (A-3), rabbit polyclonal IgG anti-GHS-R1a (H-80) and goat polyclonal IgG anti GHRL (C-18), secondary (goat anti-mouse IgG_1_-HRP conjugated and goat anti-rabbit IgG-HRP conjugated and rabbit anti-goat IgG-HRP conjugated) antibodies and protein A-Agarose) were purchased from Santa Cruz Biotechnology (Santa Cruz, CA, USA). Washing procedure was performed as described above. Proteins complexed with antibodies were detected using SuperSignal West Pico Chemiluminescent Substrate Thermo Fisher Scientific (Waltham, MA, USA 02451) according to the manufacturer’s protocol. To document equal protein loading each blot was stripped and probed with GAPDH. All presented results were obtained in six consecutive experiments.

### 4.5. Statistical Analysis

Results are expressed as means ± SEM. A statistical analysis was done by one-way analysis of variance (ANOVA), followed by Tukey’s multiple comparison test. A statistical analysis was conducted using the statistical package GraphPad Prism 5.00 (GraphPad Software, San Diego, CA, USA). Differences with *p* < 0.05 were considered significant.

## 5. Conclusions

In conclusion, ghrelin stimulates its own expression and expression of its receptor in isolated pancreatic acinar cells and AR42J cells on the positive feedback pathway. This mechanism seems to participate in the pancreatoprotective effect of ghrelin in the course of acute pancreatitis.

## Figures and Tables

**Table 1:** Gene, primers’ sequences, product length, annealing temperatures, references.

Gene	Sequence 5’ > 3’	Product	Annealing Temp. (°C)	Reference
*β-actin*	S: TTG TAA CCA ACT GGG ACG ATA TGG A: GAT CTT GAT CTT CAT GGT GCT AGG	764 bp	60	[132]
*GHS-R1a*	S: GAG ATC GCT CAG ATC AGC CAG ATC AGC CAG TAC A: TAA TCC CCA AAC TGA GGT TCT GC	313 bp	60.7	[32]
*GHRL*	S: CAG AGG ACA GAG GAC AAG CAG AAG A A: GCT GGA TGT GAG TTC TTG CTT AGG A	234 bp	59.5	[32]
